# Structural Dynamics and Activity of B19V VP1u during the pHs of Cell Entry and Endosomal Trafficking

**DOI:** 10.3390/v14091922

**Published:** 2022-08-30

**Authors:** Renuk V. Lakshmanan, Joshua A. Hull, Luke Berry, Matthew Burg, Brian Bothner, Robert McKenna, Mavis Agbandje-McKenna

**Affiliations:** 1 Department of Biochemistry and Molecular Biology, Center for Structural Biology, The McKnight Brain Institute, University of Florida, Gainesville, FL 32610, USA; 2 Department of Chemistry and Biochemistry, Montana State University, Bozeman, MT 59717, USA

**Keywords:** parvovirus B19, B19V, VP1u, receptor binding domain, PLA_2_, phospholipase, thermostability, CD spectroscopy, endosomal trafficking, structure, minute virus of mice, MVM

## Abstract

Parvovirus B19 (B19V) is a human pathogen that is the causative agent of fifth disease in children. It is also known to cause hydrops in fetuses, anemia in AIDS patients, and transient aplastic crisis in patients with sickle cell disease. The unique N-terminus of Viral Protein 1 (VP1u) of parvoviruses, including B19V, exhibits phospholipase A_2_ (PLA_2_) activity, which is required for endosomal escape. Presented is the structural dynamics of B19V VP1u under conditions that mimic the pHs of cell entry and endosomal trafficking to the nucleus. Using circular dichroism spectroscopy, the receptor-binding domain of B19V VP1u is shown to exhibit an α-helical fold, whereas the PLA_2_ domain exhibits a probable molten globule state, both of which are pH invariant. Differential scanning calorimetry performed at endosomal pHs shows that the melting temperature (T_m_) of VP1u PLA_2_ domain is tuned to body temperature (37 °C) at pH 7.4. In addition, PLA_2_ assays performed at temperatures ranging from 25–45 °C show both a temperature and pH-dependent change in activity. We hypothesize that VP1u PLA_2_ domain differences in T_m_ at differing pHs have enabled the virus to “switch on/off” the phospholipase activity during capsid trafficking. Furthermore, we propose the environment of the early endosome as the optimal condition for endosomal escape leading to B19V infection.

## 1. Introduction

Human parvovirus B19 (B19V) belongs to the genre *Erythroparvovirus* of the family *Parvoviridae* [1]. B19V was discovered in 1975 by Yvonne Cossart when evaluating serum samples for Hepatitis B antigen [2]. Later, it was shown that the virus had a narrow tropism for erythroid progenitor cells [3]. B19V infection is associated with several complications in different age and health groups; it causes hydrops in fetuses, the causative agent of erythema infectiosum in children (fifth disease), arthralgia and arthritis in adults, severe anemia in HIV-infected patients, and transient aplastic crisis in patients with sickle cell disease [4]. The transmission of B19V occurs via respiratory secretions, blood transfusions, and vertically from mother to fetus [4]. 

The B19V genome is 5.6 Kb, incorporating flanking hairpin structures that act as primers for viral DNA replication [5]. The genome encodes for the essential non-structural proteins NS1 (74 KDa), two smaller proteins of 11 and 7.5 KDa, and two capsid proteins VP1 (86 KDa) and VP2 (60 KDa). The T = 1 icosahedral capsid is assembled from VP1 and VP2 in an approximate ratio of 1:20, respectively. The VP1 and 2 share an overlapping sequence, with VP1 having an extended unique N terminus sequence, which is referred to as VP1u. The VP1u of parvoviruses have two known enzymatic activities, a secreted phospholipase A2 (sPLA_2_) and protease [6,7]. The VP1u is a group XIII PLA_2_ enzyme that has a conserved His-Asp catalytic dyad and calcium-binding loop. It has been shown that amino acid substitution of these conserved residues within the PLA_2_ domain of parvoviruses causes the virus to undergo prolonged perinuclear localization and prevents the transfer of the viral genome to the nucleus [6]. Therefore, the PLA_2_ domain is essential for successful viral infection. The amino acids responsible for the proteolytic function have not been identified, but it has been shown that changes of the His-Asp dyad of the PLA_2_ domain also cause a decrease in protease activity [7]. In addition, the VP1u of B19V has a receptor-binding domain (RBD) at the N terminus (amino acids 5–68), which is required for cell binding and internalization of the virus [8,9]. 

Parvoviruses enter the cell via receptor-mediated endocytosis and go through the endo-lysosomal pathway before entering the nucleus [10]. As they are trafficked through the endosome, acidification of the vesicles occurs, which is important for infection for several parvoviruses [11,12]. VP1u PLA_2_ activity is thought to be involved in disrupting the endosomal membrane to permit the virus to escape the endosome to the cytoplasm [6,12]. In the case of B19V, the VP1u is thought to be localized on the exterior of the viral capsid, whereas for several other parvoviruses such as adeno-associated virus (AAV) and minute virus of mice (MVM), it is internalized within the capsid [13,14,15]. It is thought that, for AAV and MVM, the VP1u only becomes externalized when the virus encounters the acidic pH conditions experienced during cellular trafficking. A study focused on understanding the dynamics of AAV1 VP1u found that the VP1u exhibits an α-helical structure and undergoes an unfolding event with acidification [16]. The authors attributed this unfolding event to aid in the externalization of VP1u during capsid trafficking. 

This study examines the dynamics of B19V VP1u under conditions that mimic the endosomal environment. Circular dichroism (CD) spectroscopy is used to monitor and demonstrate that the VP1u maintains a significant α-helical secondary structure for the entire pH range of the endosome. Further, it is shown that within the VP1u, the RBD has a significant α-helical structure, whereas the PLA_2_ domain exhibits a probable molten globular structure. Differential scanning calorimetry (DSC) experiments demonstrated that the VP1u PLA_2_ domain has a thermal melting temperature (T_m_) similar to human body temperature, and differential scanning fluorometry (DSF) experiments performed on B19V virus-like particles (VLPs) show a pH-dependent correlation between the change in thermostability of the capsid and VP1u. In addition, phospholipase activity assays show a co-dependence of VP1u PLA_2_ activity on temperature and pH. These observations suggest that the B19V capsid may have evolved to use the thermostability properties of VP1u as a structural and functional switch during capsid trafficking. Taking these observations together, we propose that the environment of the early endosome is optimal for B19V to escape the endosome and traffic to the nucleus.

## 2. Materials and Methods

### 2.1. VP1u Expression and Purification 

Three plasmids were constructed for the expression of B19V VP1u and variants. The complete VP1u gene (encoding aa 1–227) was inserted into a pET11a plasmid, while the RBD (aa 1–90), and PLA_2_ domain (aa 91–227) encoding sequences were inserted into a pET30a plasmid. The genes were derived from B19V isolate J35 (AY386330.1) and all the proteins were expressed with a C terminus 6 histidine tag. For protein expression, the plasmids were transformed into Bl21(DE3) Competent *E. coli* cells (New England Biolabs, Ipswich, MA, USA). Expression was induced in lysogeny broth media with the addition of 0.4 mM IPTG to the cell culture at an OD_600_ ~ 0.6. The cells were spun down after 3 h of induction at 37 °C, following which they were resuspended in lysis buffer (25 mM Tris-HCl, 500 mM NaCl, 10 mM Imidazole, pH 8). Cells were then lysed using the LM10 Microfluidizer (Microfluidics, Westwood, MA, USA) at 18,000 psi, and cell debris was removed by centrifugation at 12,000× *g* for 30 min at 4 °C. The supernatant was applied to 0.75 mL of Ni-NTA resin (G-Biosciences, St. Louis, MO, USA) and incubated at 4 °C for 60 min. The flow-through was discarded, and the column was washed with 20 column volumes of lysis buffer. The recombinant protein was then eluted with 5 column volumes of elution buffer 1 (25 mM Tris-HCl, 500 mM NaCl, 400 mM Imidazole, pH 8). The eluent was injected onto a HiLoad 16/600 Superdex 75 pg size exclusion column (GE Healthcare, Chicago, IL, USA), which was pre-equilibrated with storage buffer (20 mM HEPES, 100 mM NaCl, pH 7.5). The fractions pertaining to pure protein were collected, and purity was assessed by SDS PAGE. The fractions were then pooled, concentrated, and frozen at −20 °C.

Stock plasmid (pET30a) containing the MVM VP1u gene (aa 1–142), derived from J02275.1, fused to a C terminus 6 Histidine tag were transformed into Bl21(DE3) Competent *E. coli* cells (New England Biolabs, Ipswich, MA, USA). Following this, the cells were grown at 37 °C until OD_600_ ~ 0.6. The cells were cooled down to 30 °C on ice, and protein expression was induced by the addition of 0.4 mM IPTG. The cells were then grown in a shaking incubator at 30 °C for 4–5 h. Following this, the cells were spun down, resuspended in binding buffer (25 mM Tris-HCl, 500 mM NaCl, 8 M Urea, 1 mM 2-mercaptoethanol, 20 mM imidazole, pH 8), and lysed on the LM10 microfluidizer at 18,000 psi. The cell debris was removed by centrifugation at 12,000× *g* for 30 min at 25 °C. Next, the supernatant was applied to a 0.75 mL of Ni-NTA resin and incubated at 25 °C for 60 min. The flow-through was discarded and the column was washed with 50 column volumes of binding buffer. Next, the bound protein was washed with 100 column volumes of refolding buffer (25 mM Tris-HCl, 500 mM NaCl, 1 mM 2-mercaptoethanol, 20 mM imidazole, pH 8). Finally, the protein was eluted with 10 column volumes of elution buffer 2 (25 mM Tris-HCl, 500 mM NaCl, 1 mM 2-mercaptoethanol, 400 mM imidazole, pH 8). The eluent was then buffer exchanged into storage buffer (20 mM HEPES, 100 mM NaCl, pH 7.5), concentrated, and checked for purity using SDS PAGE. The concentrated samples were stored at −20 °C.

### 2.2. Expression and Purification of B19V VLPs

B19V VLPs were expressed in Sf9 cells as described previously [17]. Baculovirus stock was used to infect Sf9 cells at a multiplicity of infection (MOI) of 5 plaque-forming units (PFU). After 72 h, cells were spun down by centrifugation at 1000× *g* for 20 min at 4 °C. The supernatant was subjected to polyethylene glycol (PEG) precipitation by addition of 10% (*w*/*v*) PEG 8000 and overnight stirring at 4 °C. The PEG pellet was harvested by centrifugation at 14,300× *g* for 90 min at 4 °C and then resuspended in TNTM buffer (25 mM Tris-HCl, 100 mM NaCl, 0.2% Triton X-100, 2 mM MgCl_2_, pH 8.0). The cell pellet was also resuspended in TNTM buffer and then lysed using the LM10 Microfluidizer at 5000 psi. Following this, the lysed cells and resuspended PEG pellet were combined, benzonase treated, and centrifuged at 12,000× *g* for 30 min to remove cell debris. The clarified supernatant was loaded on 20% sucrose cushion (*w*/*v* sucrose in TNTM buffer) and centrifuged at 45,000 rpms on a Ti70 rotor for 3 h at 4 °C. The pellet obtained from the sucrose cushion was resuspended in TNTM buffer. Further, the resuspended pellet was loaded onto a 10–40% sucrose step gradient (*w*/*v* sucrose in TNTM buffer) and centrifuged at 35,000 rpm on a SW41 rotor for 3 h at 4 °C. The VLPs containing fractions were further dialyzed into phosphate-buffered saline (PBS), concentrated, and stored at 4 °C.

### 2.3. Negative Stain Electron Microscopy (EM)

Negative stain electron microscopy was used to assess the quality of B19V VLPs produced. The surface of carbon coated holey copper grids (Electron Microscopy Sciences, Hatfield, PA, USA) were first made hydrophilic by glow discharging on a PELCO easiGlow instrument. Further, 2–3 μL of the purified virus sample was loaded onto the glow discharged grids and incubated for 2 min. The grids were then washed 3 times in 20 μL water droplets, and excess liquid on the grid was blotted using a filter paper. The grid was then stained 3 times in 2% uranyl acetate and excess stain was removed by using filter paper. The grid was air dried and imaged on a 120 KeV Tecnai Spirit microscope (FEI company, Hillsboro, OR, USA).

### 2.4. Differential Scanning Calorimetry

B19V VP1u samples were buffer exchanged into a universal buffer (20 mM HEPES, 20 mM MES, 20 mM NaAc, 150 mM NaCl, 5 mM CaCl_2_) adjusted to pHs 5.5, 6.0, and 7.4 [18]. The concentration of protein was estimated by measurements at 280 nm to be between 1–2 mg/mL. A VP-Capillary DSC (Microcal, Northampton, MA, USA) instrument was used to perform the calorimetry experiments, and the universal buffer was used as the reference. Both protein and reference samples were degassed for 30 min at 15 °C before loading into the cells. Temperature ramp experiments were performed in triplicates at a scan rate of 1 °C/min from 5 to 95 °C. The thermograms were deconvoluted using the origin software suite (Microcal, Northampton, MA, USA). 

### 2.5. Differential Scanning Fluorometry

B19V VLP at 0.2–0.3 mg/mL were dialyzed into a universal buffer (20 mM HEPES, 20 mM MES, 20 mM NaAc, 150 mM NaCl, 5 mM CaCl_2_) adjusted to pH 5.5, 6.0 and 7.4 [18]. Following this, 22.5 μL of capsid was mixed with 2.5 μL of 1% SYPRO orange dye (Invitrogen, Carlsbad, CA, USA) and the assay was performed in a Bio-Rad MyiQ2 Thermocycler instrument at a ramp rate of 1 °C/min in 0.5 °C step from 30 to 99 °C. The instrument measures the rate of change of fluorescence as a function of temperature, which is plotted as −dRFU/dT vs. temperature. The values of −dRFU/dT were inverted by multiplying by −1, and the peak value on the thermogram is taken as T_m_. The experiments were performed in triplicates.

### 2.6. CD Spectroscopy

The circular dichroism (CD) spectrum of B19V VP1u and variants were obtained on an Aviv model 430 spectrometer at a sample concentration of 0.2–0.4 mg/mL. For obtaining high-quality data for secondary structure estimation, the experiments were performed in water at a wavelength range of 190–260 nm. Estimation of percent secondary structure was performed using the BeStSel algorithm [19]. For thermal unfolding and refolding studies, experiments were performed at pHs (4.0, 5.5, 6.0, 7.4) in 20 mM citrate-phosphate buffers at different temperatures. Three replicate scans were acquired for each sample within a wavelength range of 200 to 260 nm at 1 nm intervals. Average CD signal (in millidegrees) for the three scans were calculated, buffer subtracted, and the readouts were converted to Delta Epsilon (Δε) measured in M^−1^cm^−1^ by using the following formula:

Δε = θ × (0.1 × MRW)/(P × CONC) × 3298(1)

where θ is the CD signal in millidegrees, MRW is the mean residue weight, P is path length in cm, CONC is protein concentration in mg/mL.

Deconvolution of CD signal of individual domains from full-length VP1u: It was assumed that the ellipticity of the full-length protein is the sum of the ellipticity of its individual domains and that for both whole protein and each domain’s mean residual ellipticity (*MRE*) is simply the ellipticity of that component divided by the number of residues (α). *MRE_1_* is the signal from the domain being deconvoluted from both *MRE_2_* and *MRE_total_*. For each wavelength of the data, the following equation should yield the appropriate value of *MRE_1_* from the known data:

*MRE_1_* = ((*MRE_total_* × α_total_) − (*MRE_2_* × α_2_))/α_1_ (2)

### 2.7. PLA_2_ Assays

*pH profile:* For the PLA_2_ assays, 4.8 μg of B19V VP1u were incubated with 1 mM POPC liposomes (Avanti Polar Lipids, AL, USA) for 50 min in 100 mM citrate phosphate buffer, and 5 mM CaCl_2_ at pH: 4.0, 5.0, 6.0, 7.0, and 8.0. The incubated samples were then injected onto an Agilent technology 1200 infinity HPLC connected to a Bruker Daltonics MicroTOF instrument. Following injection, the chromatogram area of 496.32 m/z corresponding to a PLA_2_ cleavage product was used to determine the activity profile. All assays were performed in triplicates.

*Colorimetric assay:* In this assay 250 ng of B19V VP1u, 30 ng of MVM VP1u, and 180 ng of VP1 containing B19V particles were tested for activity using Cayman’s PLA_2_ Assay Kit (Cayman Chemical, Ann Arbor, MI, USA). The micellar substrate (diheptanoyl thio-PC) used in the assay was solubilized in pH 7.5 buffer (25 mM Tris-HCl, 10 mM CaCl_2_, 100 mM KCl, 0.3 mM Triton X-100) and pH 6.0 buffer (25 mM MES, 10 mM CaCl_2_, 100 mM KCl, 0.3 mM Triton X-100) by vortexing. Further, assays were performed using the solubilized substrate at different temperatures. The manufacturer protocol was modified to use only half the reaction volume in the assays. The absorbance was measured at 414 nm for 30 min and the slope of the linear portion of the measurements was used to calculate PLA_2_ activity based on the formula provided with the kit. The assays were performed in *n* = 6.

## 3. Results

### 3.1. Circular Dichroism Analysis Demonstrates That the Rbd (Domain 1) Is α-Helical whereas the Pla2 Domain (Domain 2) Is Predominantly Unstructured

**Figure 1:**
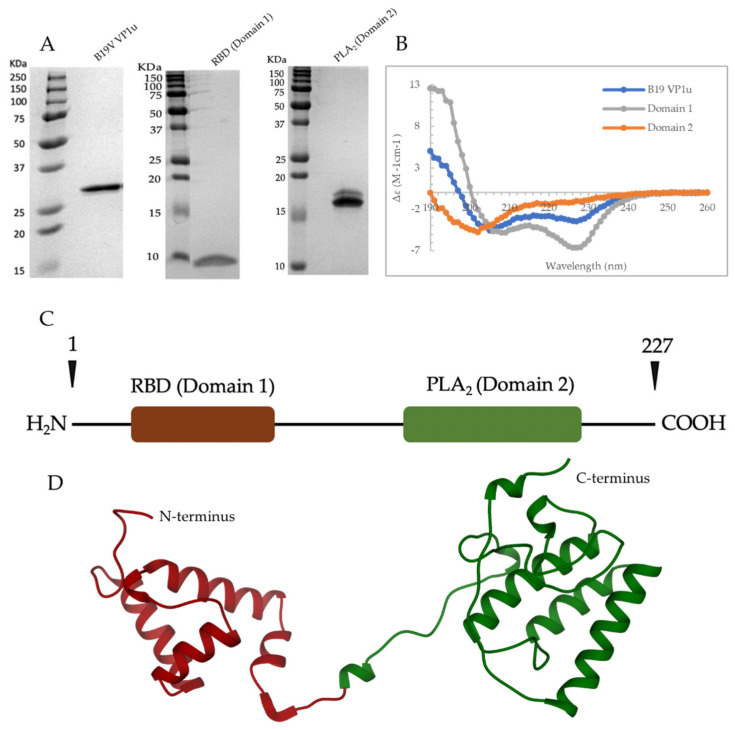
SDS PAGE and structure of B19V VP1u. (**A**) SDS PAGE profile of B19V VP1u, domain 1, and domain 2. (**B**) Representative CD spectrum of B19V VP1u, RBD (domain 1), and PLA_2_ (domain 2) recorded in water at 20 °C. (**C)** Schematic of B19V VP1u with the RBD (dark brown) and PLA_2_ domain (green). (**D**) In silico model of B19V VP1u generated using RoseTTAFold with the RBD (dark brown) and PLA_2_ domain (green).

To study the secondary structure of B19V VP1u, CD experiments were performed on the full-length protein and the RBD (domain 1) and PLA_2_ (domain 2) domains separately. The CD spectrum of full length B19V VP1u shows a dominant α-helical signal (Figure 1B). The helical nature of VP1u was expected since crystal structures of several PLA_2_ enzymes including snake venom and bee-venom PLA_2_ show a primarily α-helical structure [20,21]. In addition, the RBD identified within VP1u has also been predicted to be α-helical (Figure 1C,D) [8]. Additionally, an in silico model of B19V VP1u calculated using RoseTTAFold predicted a predominately α-helical structure (Figure 1D) [22]. However, an estimation of the percent secondary structure elements shows that VP1u has approximately 30% α-helical content (Table 1). The remainder consists of β-sheet (10%) and turns or disordered regions (60%). This suggests that the calculated in silico models may not be accurately describing the secondary structure of VP1u in vitro. Considering there are two possible α-helical domains within the protein, the observed CD signal may correspond to either one of them or both. Therefore, to assign the origin of the α-helical signal, two deletion variants of B19V VP1u were generated. One, domain 1, encoding amino acids 1–90 containing the predicted RBD, and the other, domain 2, encoding amino acids 91–227 containing the PLA_2_ domain. Further CD analysis on domain 1 and 2, clearly demonstrated that the RBD exhibited the α-helical structure, whereas the PLA_2_ domain exhibited a predominantly disorder structure (Figure 1B). This showed that the α-helical signal observed for B19V VP1u has a major contribution from RBD. Consistent with the CD spectrum, estimation of secondary structure elements shows that domain 1 (RBD) has 40% α-helical content and 60% turns or disordered region. The estimation of percent secondary structure elements within domain 2 (PLA_2_ domain) shows that there is only 10% α-helical content, with 25% β-sheet and 65% turns or disordered regions (Table 1). This suggests that although the PLA_2_ domain is predominantly disordered, it still has some structural features.

**Figure 2:**
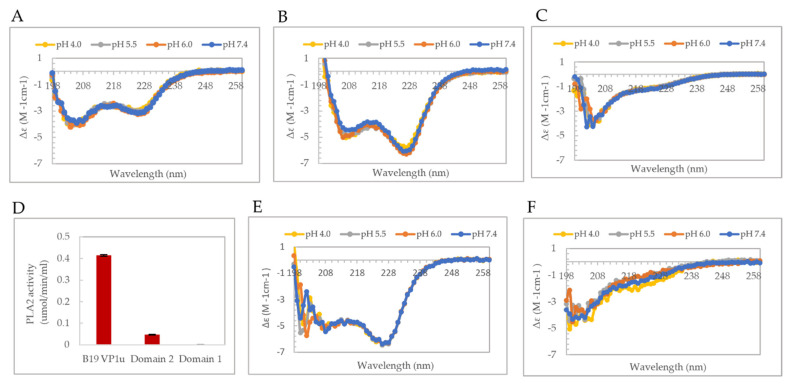
PLA_2_ activity and pH dependence of secondary structure. (**A**–**C**) Representative CD spectrum of B19V VP1u (left), RBD (domain 1, middle) and PLA_2_ domain (domain 2, right) at pH 4.0, 5.5, 6.0, and 7.4 measured at 20 °C. (**D**) PLA_2_ activity of 250 ng of B19V VP1u, domain 2 and domain 1. Calculated CD spectrum of RBD (**E**) and PLA_2_ domain (**F**) at different pHs.

Furthermore, to study the effect of pH on the secondary structure of B19V VP1u and the individual domains, CD experiments were performed at pHs 4.0, 5.5, 6.0, and 7.4. The observed α-helical signal of B19V VP1u seems to be unaffected by a change in the pH in the range of endosomal trafficking (Figure 2A). Consistent with the CD spectrum of B19V VP1u, no secondary structural change was observed with a change in pH for either domain 1 and 2 (Figure 2B,C). This indicates that B19V VP1u is unlikely to undergo pH-related conformational changes when it experiences acidic environments. 

### 3.2. Phospholipase A2 Activity of Domain 2 Is Affected by the Presence/Absence of the RBD (Domain 1)

Following the secondary structure assignment of domain 1 and 2, a PLA_2_ assay was performed on B19V VP1u and the individual domains. Interestingly, the PLA_2_ activity of domain 2 was seen to be 10-fold lower when compared to full length B19V VP1u (Figure 2D). This suggests that the presence of the RBD is required for optimal PLA_2_ enzyme activity. Since all the conserved residues required for PLA_2_ activity are present within domain 2, the loss of function could be attributed to a structural alteration of VP1u associated with the absence of the RBD. As domain 2 showed a decrease in function and is predominantly disordered, the next question asked was, does the PLA_2_ domain (domain 2) have a more ordered structure within the context of full-length B19V VP1u? To address this question, the CD signal contribution of the PLA_2_ domain from B19V VP1u were algebraically deconvoluted (refer to Section 2.5). The result clearly shows that the PLA_2_ domain is predominantly disordered within the context of B19V VP1u as well (Figure 2F). Similarly, the deconvolution of the signal contribution of RBD from B19V VP1u resulted in an α-helical spectrum comparable to that of domain 1 only (Figure 2B,E). These results confirm that the overall secondary structure of both domains within B19V VP1u are conserved within the expressed deletion variants. The results also suggested that the RBD (domain 1) and PLA_2_ domain (domain 2) fold independently of each other, but both are required for optimal PLA_2_ activity. This independent folding seems plausible considering that the RBD and PLA_2_ domain are connected via a long flexible-linker region (53 amino acids) (Figure 1D). However, it is not clear if the unstructured nature of the PLA_2_ domain is conserved within the viral capsid or if it is an artifact of in vitro expression of VP1u.

### 3.3. Ph-Induced Variation in Thermostability of Viral Capsid Correlates with That of VP1u

**Figure 3:**
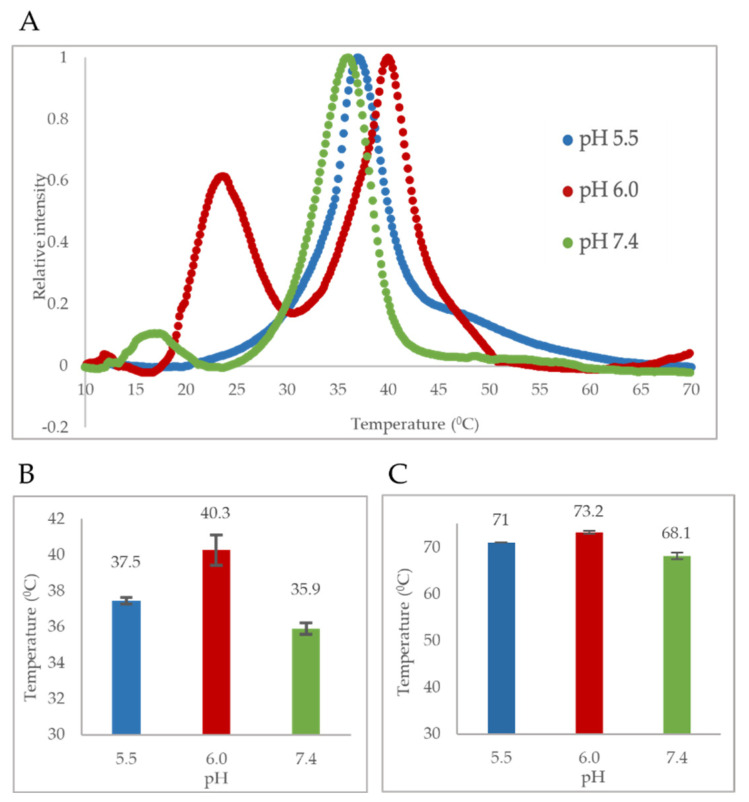
Thermostability of B19V capsid protein. (**A**) Comparison of experimental baseline corrected DSC thermograms of VP1u at different pHs. (**B**) pH dependent thermostability profile of B19V VP1u based on the major endothermic peak. (**C**) pH dependent thermostability profile of B19V VLPs. All experimental values are shown as means ± standard deviation (*n* = 3).

Both DSF and DSC methods were used to obtain a pH-dependent thermostability profile of B19V VLPs and VP1u, respectively. Shown are the pH profiles obtained for VP1u based on the major endothermic peak present in the DSC thermograms (Figure 3A,B). The profile shows that the T_m_ of this unfolding intermediate at pH 7.4 is human body temperature, indicating that, when the virus encounters physiological conditions (pH 7.4 and 37 °C), it could be unfolding. Furthermore, an increase in the T_m,_ due to acidification could indicate that when the B19V capsid encounters acidic conditions, the unfolding intermediate could refold. The DSC thermograms recorded at different pHs also show the presence of several endothermic transition peaks (Figure 3A). The lack of a single symmetrical peak suggests that the unfolding of VP1u does not fit a cooperative two-state model. It appears to proceed via multiple unfolding intermediates. This is not surprising because we have shown that VP1u contains at least two functional domains, which fold independently of each other (Figure 2). These intermediates may be representing the RBD and PLA_2_ domain. The major peak is likely not representing the RBD because it cannot be unfolded at physiological conditions, as it is required for cell entry. Therefore, it could be concluded that the major peak transition is the PLA_2_ domain, which is also the larger functional domain within VP1u. 

On comparing the pH-dependent thermostability profiles of VP1u and VLPs, it is apparent that both have higher thermal stability at pH 6.0, which is decreased at pH 7.4 and 5.5 (Figure 3B,C). Furthermore, there is a correlation between the change in thermostability of VP1u and VLP at these pHs as an increase in pH from 5.5 to 6.0, causes a 2.8 °C increase in T_m_ of VP1u and a 2.2 °C increase in T_m_ of VLPs. Similarly, an increase in pH from 6.0 to 7.4 causes a 4.4 °C decrease in T_m_ of VP1u and 5.7 °C decrease of VLPs. The positive correlation could mean that both the VP1u and full length VP1, including the capsid β-barrel, have co-evolved to attain higher thermostability at acidic pH. This could be beneficial for the virus by providing increased protease resistance within the endosomal compartment. This observation of a correlation between protease resistance and thermostability has been reported in the literature for other proteins [23,24]. Therefore, evolving higher thermostability of the capsid and VP1u at low pH may have advantages for enhanced infectivity of these viruses.

### 3.4. VP1u Undergoes Thermal-Induced Unfolding and Refolding

**Figure 4:**
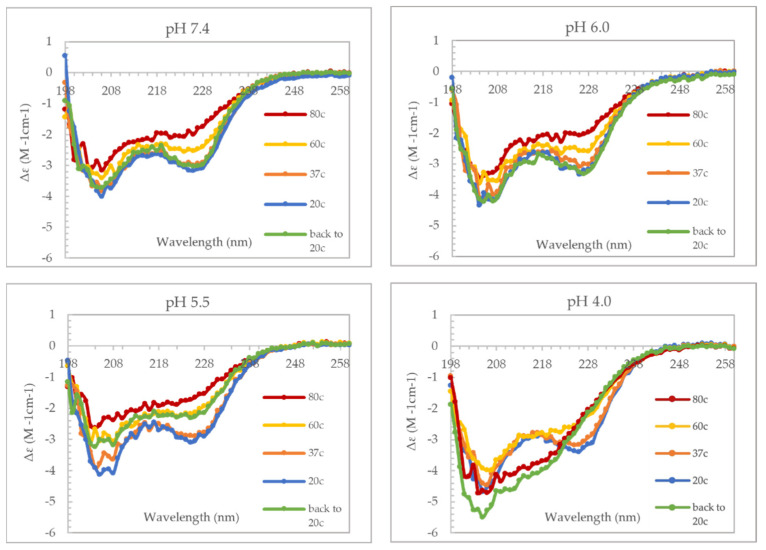
Effect of heat on secondary structure of B19V VP1u. The CD spectrum was recorded at pHs 4.0, 5.5, 6.0 and 7.4.

The observations from the DSC experiments suggest that VP1u can undergo thermal-induced conformational changes when the protein encounters the acidic pHs of the endosomes. This led to the hypothesize that B19V VP1u might undergo thermal-induced unfolding and refolding during cellular trafficking. To study thermal transitions and the effect of pH on secondary structure of B19V VP1u, CD spectroscopy was used to perform temperature ramping experiments at pHs 7.4, 6.0, 5.5, and 4.0 (Figure 4). Not unexpectedly, the data clearly demonstrated a loss of α-helical signal with temperature increased to 80 °C at the four pHs tested. However, complete reversible folding of the protein, on returning the temperature to 20 °C, was observed only for pHs 7.4 and 6.0. At pH 5.5, the protein precipitated on cooling, which is the reason for the reduction in α-helical signal. While at pH 4, cooling to 20 °C resulted in a significant change in the CD spectrum suggesting possible misfolding of the protein. Thermal-induced unfolding and refolding prove that VP1u can undergo conformational changes at pHs of physiological relevance.

Interestingly, there was minimal loss of α-helical signal at 37 °C at all the tested pHs despite DSC data suggesting a major increase in heat capacity at this temperature. Already established from previous data, the α-helical signal of VP1u is mainly contributed from the RBD (Figure 2). These two observations taken together, therefore suggests that the RBD has a higher melting temperature and that it is folded at 37 °C. To further confirm this hypothesis, similar thermal-induced unfolding and refolding experiments were performed on domain 1 (RBD). This experiment yielded a similar result, which shows a temperature-dependent unfolding and refolding of the RBD at pHs 7.4, 6.0, and 5.5, but not at pH 4.0 (Supplementary Figure S1). The experiment furthermore confirms that the RBD maintains its secondary structure at 37 °C at all the tested pHs. This makes sense when considering the life cycle of the virus, because the virus needs the RBD to always be folded to be able to bind its cellular receptor. 

### 3.5. VP1u Shows Temperature and pH-Dependent PLA_2_ Activity

**Figure 5:**
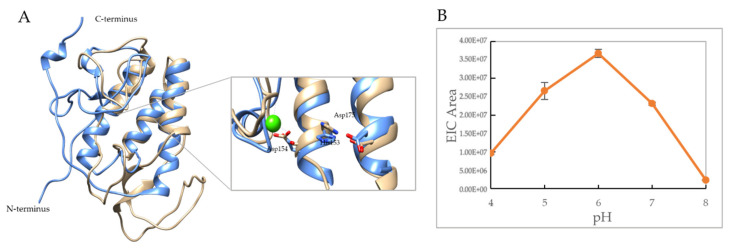
PLA2 catalytic residues and activity. (**A**) Structure superposition of model of B19V PLA2 domain (blue) on Bee venom PLA2 crystal structure (color- beige, Cα rmsd = 1.1 Å, PDB- 1POC). VP1u PLA2 catalytic residues His153 and Asp175 are highlighted. Asp154 is predicted to bind calcium (Green sphere). (**B**) Plot shows pH dependence of PLA2 activity measured at 25 °C. All experimental values are shown as means ± standard deviation (*n* = 3).

The VP1u of parvoviruses has a group XIII secreted PLA_2_ (sPLA_2_) enzyme domain, and has the conserved His-Asp catalytic dyad and require calcium for activity [6,25]. A structure superposition of the B19V VP1u in silico model on the crystal structure of Bee venom PLA_2_ reinforces this conservation, with the presence of a conserved three-helix bundle and a calcium-binding loop characteristic of sPLA_2_ enzymes (Figure 5A). The positions of the His-Asp catalytic dyad and residues involved in binding calcium are conserved between the structures, thus implying the validity of the predicted structural model of the B19V VP1u. Furthermore, experimental results from a pH-dependent PLA_2_ activity profile obtained by assaying B19V VP1u with phosphatidylcholine liposomes at 25 °C, show optimum phospholipase activity at pH 6.0 with activity decreasing at more basic or acidic pHs (Figure 5B). This information reaffirms data that has previously been reported in the literature [25]. 

**Figure 6:**
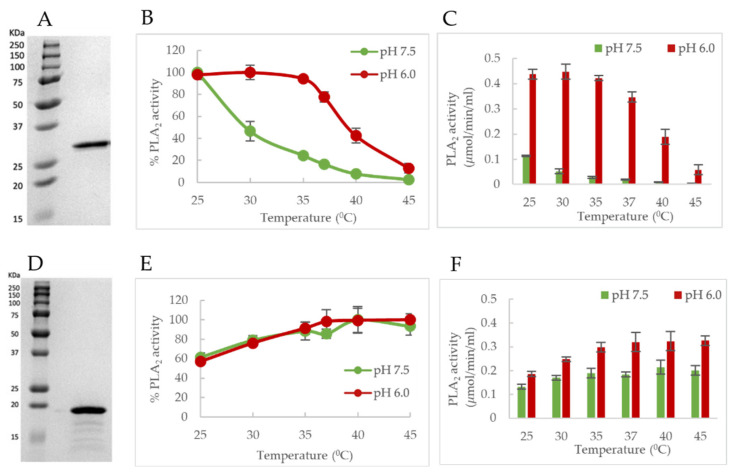
Effect of temperature and pH on VP1u PLA2 activity. (**A**) SDS PAGE of B19V VP1u. (**B**) PLA2 activity of B19V VP1u (250 ng) plotted as relative maximum activity. (**C**) PLA2 activity of B19V VP1u quantified at different temperatures and pHs. (**D**) SDS PAGE of MVM VP1u. (**E**) PLA2 activity of MVM VP1u (30 ng) plotted as relative maximum activity. (**F**) PLA2 activity of MVM VP1u quantified at different temperatures and pHs. All experimental values are shown as means ± standard deviation (*n* = 6).

We proposed that the major peak in the DSC thermogram is contributed by the PLA_2_ domain (Figure 3B). If this is correct, a loss of PLA_2_ activity should be observed with an increase in temperature. Therefore, to study the temperature dependence of VP1u PLA_2_ activity, assays were performed at increasing temperatures from 25 to 45 °C on B19V and Minute virus of mice (MVM) VP1us. The assay performed on B19V VP1u shows that the PLA_2_ activity was optimal at 25 °C, at the tested pHs and activity was higher at pH 6.0 when compared to pH 7.5 (Figure 6B,C). When assayed at increasing temperatures, B19V VP1u showed a temperature and pH-dependent decrease in PLA_2_ activity. At pH 7.5 and 37 °C, enzyme activity was reduced to 17% relative to activity at 25 °C. This reduction can be explained by considering the T_m_ of the PLA_2_ domain at pH 7.4 which is 35.9 °C. As the temperature approaches 37.0 °C, there is an observed steep decrease in activity, likely caused by the unfolding of the PLA_2_ domain resulting in low-level activity at 45 °C. Even at pH 6.0, the enzyme activity follows a similar temperature dependence. The activity remained relatively high until 35 °C, but as the temperature approached T_m_ (40.3 °C), the activity starts to decrease with the enzyme retaining 78% relative activity at 37 °C and finally baseline level activity at 45 °C. 

This experiment was also conducted on another parvoviral phospholipase, MVM VP1u, to observe the co-dependence on temperature and pH for PLA_2_ activity, MVM is an infectious agent of laboratory mice (Figure 6E,F). The enzyme activity of MVM VP1u peaked at 37 °C as opposed to B19V VP1u, for which peak activity was observed at 25 °C. Further, the activity of MVM VP1u did not decrease on additional heating, until 45 °C at both the tested pHs. This suggests that the T_m_ of MVM PLA_2_ domain is >45 °C at the tested pHs. Comparison of activity at pH 7.5 and 6.0 shows that activity is higher at pH 6.0 (Figure 6F). The data shows a clear distinction in the temperature and pH dependence of B19V and MVM VP1u PLA_2_ activities. This suggests that different parvoviruses may have evolved unique mechanisms to alter VP1u dynamics during capsid trafficking.

### 3.6. B19v Vlps Shows Temperature and Ph-Dependent Pla2 Activity

**Figure 7:**
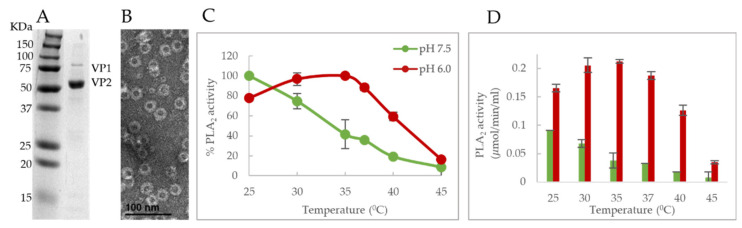
Effect of temperature and pH on PLA2 activity of B19V VLPs. (**A**) SDS PAGE of purified VLP showing VP1 and VP2. (**B**) Negative-stain EM showing assembled virions. (**C**) PLA2 activity of B19V VLPs (180 ng of VP1) measured at different temperatures and pHs plotted as relative maximum activity. (**D**) Quantification of PLA2 activity of B19V VLPs at different pHs. All experimental values are shown as means ± standard deviation (*n* = 3).

Based on these observations, the next question we asked was, does the location of the VP1u domain, within the context of the viral capsid exhibit a similar temperature and pH-dependent cleavage of phospholipids? To test this hypothesis, PLA_2_ assays were performed on B19V VLPs at temperatures ranging from 25–45 °C at pHs 6.0 and 7.5. As expected, B19V VLPs showed an activity phenotype comparable to VP1u (Figure 7C,D). Peak PLA_2_ activity was observed close to 25 °C and activity decreased as experimental conditions approached the melting temperature of the PLA_2_ domain at the respective pHs. At pH 7.5 and 37 °C, enzyme activity was reduced to 36% relative to activity at 25 °C whereas, at pH 6.0 and 37 °C, the activity was reduced to 88%. The overall trend observed in activity remained similar between VP1u and VLPs. It should be noted that the reason for VP1u showing significantly higher activity towards substrate in comparison to VLPs is due to the usage of a higher concentration of VP1u for the assay.

The existence of a similar PLA_2_ activity phenotype, which is co-dependent on pH and temperature in both B19V VP1u and VLPs suggests that this observation may be significant in the context of the virus lifecycle. This phenomenon can be explained when considering the localization of VP1u on the viral capsid. In B19V, the VP1u is localized on the exterior of the capsid. Therefore, lower activity at pH 7.5 and 37 °C might be beneficial for the virus before cell entry to prevent any cytotoxic effects. Further, an increase in activity at pH 6 and 37 °C could be important for endosomal escape as the virus traverses the endo-lysosomal pathway.

## 4. Discussion

Secreted phospholipases are a family of enzymes that uses a conserved His-Asp catalytic dyad to cleave phospholipids. These enzymes are also characterized by a need for millimolar concentrations of calcium for activity and the presence of a large number of disulfide bonds [26]. Parvoviral PLA_2_ enzymes belong to group XIII within the sPLA_2_ family. They are different from conventional sPLA_2_ enzymes because they lack cysteines, and therefore the disulfide bonds which usually confer a high level of thermal/pH stability for these enzymes. The absence of disulfide bonds may have been critical in the evolution of parvoviral PLA_2_ enzymes, to provide more conformational dynamics in the context of the viral capsid location and cellular trafficking. 

The VP1u of parvovirus B19, a sPLA_2_ enzyme, is unique among other parvoviral PLA_2_s due to its unusual length and also its external localization on the surface of the viral capsid [27]. The presence of an N terminal extension that also contains a RBD is the reason for the longer length of the B19V VP1u, compared to other parvoviruses (Figure 1C,D). It has been shown that the RBD is sufficient and required for entry of these viruses into permissive cells [8]. The RBD has been shown to have a role in the restricted tropism of B19V because the expression of the VP1u receptor has been found to be limited to erythroid progenitor cells, which are the cells infected by B19V [28,29]. In the past, globoside was proposed to be the primary entry receptor for B19V; however, recent studies have proved otherwise [30]. It has been shown that B19V can internalize into globoside knockout cells, proving the irrelevance of globoside in cellular entry [31,32]. Instead, it has been found that B19V interaction with globoside occurs within the early endosomal vesicles (~pH 6) [31,32]. However, the role of globoside in the viral life cycle is not clear.

The C terminus of B19V VP1u constitutes the PLA_2_ domain, which has an HDXXY motif and also a YXGXG motif, both of which are conserved in other sPLA_2_ enzymes [6]. Even though parvoviral PLA_2_s were identified 20 years ago now, no structural information is available so far. Circular dichroism studies performed on AAV capsids previously have shown that AAV VP1u exhibits an α-helical fold [16]. To study the secondary structure of B19V VP1u, CD experiments were performed on a full-length protein and its individual domains, RDB (domain 1) and PLA_2_ (domain 2). The CD spectrum of full-length VP1u shows that the protein has a core α-helical fold, which agrees with the predicted structure model (Figure 1B,D). However, deconvolution of the CD signal estimates only 30% α-helical content within the protein (Table 1). The remainder is β-sheets (10%) and turns or disordered regions (60%). This led us to express the RBD (domain 1) and PLA_2_ (domain 2) domains as individual variants to identify the origin of the α-helical signal. Moreover, additional CD experiments performed on these variants demonstrated that the RBD is the primary source of the α-helical signal and the PLA_2_ domain lacks an ordered structure (Figure 1B). Furthermore, an activity assay revealed that domain 2 shows a 10-fold decrease of PLA_2_ function in the absence of the RBD (Figure 2D). This is suggesting that the presence of the RBD is essential for PLA_2_ function. To understand if the loss of PLA_2_ activity is due to a structural alteration of domain 2, we performed a deconvolution of the signal contribution of the PLA_2_ domain and RBD from the full-length B19V VP1u CD spectrum. The results show that the overall secondary structure of both the functional domains of B19V VP1u are conserved within the two variants (Figure 2E,F). This confirms that the PLA_2_ domain is unstructured within full-length VP1u. However, a secondary structure estimation based on the CD spectrum of domain 2 shows that the protein has 10% α-helical content. The presence of structural components within the PLA_2_ domain was also confirmed by the major endothermic peak observed in DSC thermograms. We showed that the major peak observed close to 37 °C corresponds to the PLA_2_ domain (Figure 3A). Considering that an increase in heat capacity has a major contribution from the hydration of buried hydrophobic residues, we could infer that the PLA_2_ domain has some amount of folded structure. However, CD spectroscopy shows that this is not a well-defined structure and that it is dominated by unstructured regions. Therefore, this led us to hypothesize that the PLA_2_ domain might be in a partially folded state, also referred to as molten globule. If the PLA_2_ domain does exhibit in a molten globule conformation, this could imply that the protein could become more ordered in response to a range of external stimuli, which might include change in pH or temperature, presence of a binding partner, presence of a natural ligand, etc. However, more experiments are required to confirm this hypothesis. In addition, no change in secondary structure was observed when CD experiments were performed at different pHs (Figure 2A,C). It is not clear if the PLA_2_ domain can attain a more ordered state or if it remains disordered when it performs its function. More structural experiments are required before we can make conclusions about the native state of the B19V VP1u PLA_2_ domain. 

Previous studies have shown that the VP1u of several parvoviruses including canine parvovirus (CPV), MVM, and adeno-associated virus (AAV) only become externalized and accessible to antibodies after treatment with heat or urea. This data is suggestive of an internal localization of VP1u within the viral capsid that only becomes externalized when subjected to a change in environmental conditions [33,34,35]. In addition, CD experiments have reported that the VP1u of AAVs undergo a loss of α-helical signal when the capsid is subjected to a decrease in pH from 7.5 to 4.0 [16]. The authors attributed this loss in signal to possible unfolding and externalization of the VP1u as the virus experiences lowering pH, mimicking the environment of the endo-lysosomal pathway [16]. 

The B19V is different from these parvoviruses, in that its VP1u is permanently located on the exterior of the capsid [27]. Our CD studies show there are no pH-dependent changes in the secondary structure of B19V VP1u as well as the individual RDB and PLA_2_ domains did not show any changes in secondary structure on acidification (Figure 2A–C), unlike AAV [16]. This difference in the dynamics of B19V VP1u, compared to AAV VP1u was not unexpected, considering the difference in localization of the protein relative to the viral capsid. The lack of change in secondary structure suggests that B19V VP1u does not undergo a complete unfolding event during capsid trafficking. It has been previously shown that VP1u on native B19V capsid becomes accessible to antibodies only on receptor binding or by exposure to heat/low pH [13,36]. This suggests that a conformational change of the viral capsid is required for exposure of VP1u during cell entry. However, the virus does not experience low pH during receptor binding and, therefore, any conformational changes of the viral capsid occurring in the extracellular space cannot be triggered by pH. The role of soluble globoside in increasing B19V VP1u externalization on viral capsid has been shown previously [36]. The data we present suggest that in addition to globoside, body temperature has a role in causing conformational changes “switches” of VP1u during capsid trafficking (Figure 6B,C). 

The pH-dependent thermostability profile of B19V VP1u shows that the PLA_2_ domain has a T_m_ closely resembling human body temperature (37 °C) (Figure 3A,B). Interestingly, the least thermostability was observed at pH 7.4 (T_m_ = 35.9 °C). This led us to hypothesize that the PLA_2_ domain is most likely in a thermally “unfolded state” at 37 °C at pH 7.4, which mimics the conditions of the extracellular environment. It has been shown previously that VP1u is cytotoxic to cells, which is due to its inherent PLA_2_ activity [37]. Therefore, maintaining the VP1u in an ‘unfolded state’ could be advantageous for the virus in preventing or lowering any cytotoxic effect on the host, considering the external localization of VP1u on the viral capsid. Besides, the virus does not need the PLA_2_ activity during the cell entry process. The low T_m_ value of the PLA_2_ domain further led us to hypothesize that enzymatic activity will decrease as experimental conditions approach 37 °C. As expected, studies show a temperature-dependent change of PLA_2_ activity (Figure 6B,C). At pH 7.5, optimum activity was observed at 25 °C with a further increase in temperature resulting in loss of activity as assay conditions approached the T_m_. At 37 °C, the enzyme retained only 17% of its PLA_2_ activity relative to 25 °C, followed by baseline activity at 45 °C. The loss of activity with an increase in temperature is most likely caused by the lower thermostability of the PLA_2_ domain at pH 7.5. These observations suggest that the PLA_2_ domain of B19V has evolved, such that its structure can be modulated by pH and temperature. Further, the virus could be using this dynamic nature of VP1u to keep its inherent PLA_2_ activity “switched on/off” when the virus needs/does not need to use it.

**Figure 8:**
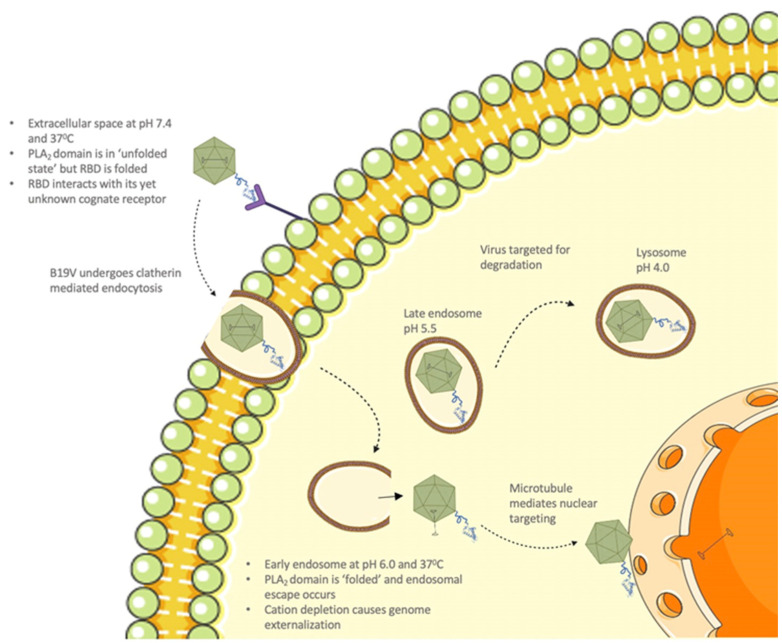
Proposed model for internalization and cellular trafficking of parvovirus B19. Made using Servier medical ART (https://creativecommons.org/licenses/by/3.0/). Accessed on 20 May 2021.

It is known that, upon interaction with its cellular receptor, B19V undergoes clathrin-mediated endocytosis [12]. Following internalization, the virus co-localizes with the early endosome, late endosome, and lysosomes (Figure 8) [12]. Furthermore, it has been shown that B19V requires acidification for endosomal escape and also found that endosomal acidification immediately after internalization might be advantageous for infection [12]. The thermostability profile of VP1u shows that the T_m_ of the PLA_2_ domain increases to 40.3 °C at pH 6.0. This suggests that VP1u can alter its structure conformation to a “folded (active) state” when the virus encounters conditions mimicking the early endosome. Further, temperature-dependent PLA_2_ activity profile at pH 6.0 shows that optimum activity was observed at 25 °C, which remained consistent until assay conditions reached 37 °C. At 37 °C, the enzyme retained 78% activity relative to 25 °C, followed by the appearance of low-level activity at 45 °C. Moreover, the pH-dependent activity profile shows the highest activity at pH 6.0 compared to other pHs (Figure 5B and Figure 6C). These observations support the idea that VP1u is in a folded and functional state when the virus encounters early endosomal conditions. Previous studies have shown that a portion of the incoming virions is routed to the late endosomes (pH 5.0–5.5) followed by lysosomes (pH 4.6–5.0) for degradation [12,38]. The thermostability profile of VP1u shows that acidification to pH 5.5 results in a decrease in T_m_ to 37.5 °C. This suggests that VP1u could revert to an “unfolded (inactive) state” as the virus progresses through the endocytic pathway (Figure 8). Besides, the pH-dependent activity profile of VP1u shows a significant decrease in PLA_2_ activity at pH 5.0 and 4.0 (Figure 5B). These observations indicate that acidification below pH 6.0 causes a decrease in thermostability and PLA_2_ activity. Therefore, it seems unlikely that the virus can utilize its inherent PLA_2_ activity to escape the endosome at pH < 6.0. It is known that B19V interacts with globoside within the early endosomal vesicles [32]. Considering that a direct interaction between VP1u and globoside has not been established yet, it is possible that the model presented in this manuscript works independent of capsid interactions with globoside. 

Taking these observations together, we propose that the PLA_2_ activity of B19V is “switched on” after internalization into the early endosome, at which point, the virus escapes into the cytoplasm (Figure 8). Following endosomal escape, the pH reverts to 7.4 within the cytoplasm, which could cause the PLA_2_ domain to be in an unfolded state (switched off). Once within the cytoplasm, additional data suggests that B19V uncoating could occur, following which the virus undergoes microtubule-mediated retrograde transport to the nucleus [39]. For our model to be valid, there are some caveats to consider, first VP1u should be able to undergo thermal and pH-induced unfolding and refolding. Secondly, the RBD should be folded when the virus is in the extracellular environment for the virus to be able to bind the VP1u receptor. To demonstrate if VP1u can unfold and refold at different pHs, we used CD spectroscopy to perform a temperature ramp experiment (Figure 4). The results from this experiment show that VP1u undergoes an unfolding event with an increase in temperature, as is evident from the loss of α-helical signal at 80 °C. Furthermore, cooling the protein back to 20 °C shows complete refolding at pHs 7.4 and 6.0 both. The absence of refolding at pH 5.5 can be explained by precipitation of sample on cooling back to 20 °C. While at pH 4.0, there is a change in the secondary structure on cooling to 20 °C, suggesting misfolding of the protein. The CD data furthermore shows that there is minimal loss of the α-helical signal at 37 °C, suggesting that the RBD is folded at all the pHs tested (Figure 4). This was confirmed by performing a similar set of CD experiments on the variant domain 1 (Supplementary Figure S1). The results are comparable to the unfolding and refolding events observed for full-length VP1u. The RBD showed minimal loss of α-helical signal at 37 °C suggesting that it has a higher melting point compared to the PLA_2_ domain. The higher thermostability of RBD means that it is intact during viral entry and endo-lysosomal trafficking which implies that its interaction with the VP1u receptor is most likely unaltered when the virus is within the extracellular space. However, changes in the spatial organization and conformation of VP1u due to its interaction with the VP1u receptor is a possibility which requires additional experimentation to understand. Furthermore, the outcome of these experiments supports the proposed model for the endosomal escape mechanism of B19V.

Previous studies examining the thermostability of AAV capsids have shown an increase in thermostability of the capsid protein with acidification [40]. The reason for such a phenomenon remains to be identified. The pH-dependent thermostability profile of B19V VLPs also shows an increase in thermostability with acidification, until pH 6.0, when an inflection point is reached (Figure 3C). One advantage of such an increase in capsid stability could be improved protease resistance within endosomal compartments. It has been described in the literature that there is a general correlation between protease resistance and the thermostability of proteins [23,24]. It is also interesting to note that there is a correlation between the change in thermostability of VP1u and VLPs with the change of pH. This could be indicative that VP1u and the jelly roll motif folds may have co-evolved to achieve higher thermostability at lower pHs. We demonstrated that an increase in thermostability at low pH is also beneficial for the PLA_2_ activity. Similarly, it could be beneficial for the viral capsid by improving protease resistance. Taken together, the data suggests that higher thermostability of the jelly roll motif and VP1u at low pHs may have been an important step in the evolution of these viruses to improve infectivity. 

We also showed that the PLA_2_ activity profile was similar for both *E. coli* expressed VP1u and sf9 expressed VLPs. We found that B19V VLPs exhibit a similar alteration of PLA_2_ activity in a pH and temperature-dependent manner, and the optimum activity was found to be close to 25 °C at both the tested pHs (Figure 7C,D). Any additional increase in temperature resulted in a decrease in activity like VP1u and reached baseline levels at 45 °C. This adds confirmation to the hypothesis that a functional “switching” of VP1u occurs on the viral capsid and it is co-dependent on the pH and Tm. 

To our knowledge, the temperature dependence of PLA_2_ activity has not been explored for any of the members of the family *Parvoviridae*. This led us to investigate the presence of a similar phenotype in other parvoviruses. We performed a similar assay on the VP1u of MVM to obtain a temperature and pH-dependent PLA_2_ activity profile. As opposed to B19V, the VP1u of MVM is smaller (142 amino acids) and located within the viral capsid. The VP1u of MVM is thought to externalize when the virus encounters suitable conditions during capsid trafficking [33].

We found that the temperature and pH-dependent PLA_2_ activity profile of MVM VP1u differs compared to that of B19V VP1u (Figure 6E,F). The optimum temperature for phospholipase activity was shown to be 37 °C and activity remained relatively high until 45 °C. This was the case at both the tested pHs and interestingly, the lowest PLA_2_ activity was observed at 25 °C in contrast to B19V, which showed the highest PLA_2_ activity at 25 °C. Additionally, activity remained relatively high at 45 °C, whereas B19V showed the lowest PLA_2_ activity at 45 °C. A comparison of PLA_2_ enzyme activity at pH 6.0 and 7.5 shows higher activity at pH 6.0, like B19V VP1u. This data suggests that the co-dependence of VP1u PLA_2_ activity on temperature and pH is observed in other parvoviruses, but the overall phenotype varies. This difference in the active/pH profile phenotype could be influenced by the differential localization of VP1u on different parvoviruses. For instance, it may not be essential for MVM to maintain low PLA_2_ activity at pH 7.5 due to the internal localization of VP1u. As being internalized in the capsid would prevent MVM VP1u from interacting with cellular phospholipid at the wrong moment during the infection process. Consequently, this protein has not evolved a temperature-dependent functional “switch” for modulation of activity. The identification of such a unique phenotype in B19V VP1u and its absence in MVM VP1u suggests that different parvoviruses have evolved different mechanisms to utilize the dynamic nature of this enzyme. In addition, it seems that the relative size of VP1u of parvoviruses could dictate the localization of these proteins within the context of the capsid. For parvoviruses with relatively low molecular weight VP1u domains (for example AAV, and MVM), an internal localization would suffice to keep the PLA_2_ domain from unwanted immune responses. Alternatively, in parvoviruses with large molecular weight VP1u domains (for example B19V), these proteins are externalized on the surface of the capsid due to a lack of space within the capsid interior. Furthermore, evolutionary pressure due to the external localization of the PLA_2_ domain on the viral capsid could have caused the VP1u of B19V to evolve a temperature- and pH-dependent switch to alter its functionality during capsid trafficking.

This study describes the structure of B19V VP1u and demonstrates the role of T_m_ pH sensitivity in the structural dynamics of the PLA_2_ enzymatic domain. We have also identified that the VP1u of MVM shows a PLA_2_ activity phenotype, which is different from B19V VP1u. Understanding the role of VP1u in the life cycle of parvoviruses is significant considering the importance of this enzyme in infectivity.

## Figures and Tables

**Table 1:** Estimated secondary structure content (%) in water.

	VP1u (1–227)	RBD (1–90, Domain 1)	PLA_2_ (91–227, Domain 2)
α-helix	20	40	10
β-sheets	10	0	25
Others	60	60	65

## Data Availability

Not applicable.

## Supplementary Materials

The following supporting information can be downloaded at: https://www.mdpi.com/article/10.3390/v14091922/s1, Figure S1: Effect of heat on the secondary structure of the RBD.
