# Supplementary material for: Structural Dynamics and Activity of B19V VP1u during the pHs of Cell Entry and Endosomal Trafficking
Source: Viruses. 2022 Aug 30;14(9):1922. doi: 10.3390/v14091922 (PMC9505059; doi:10.3390/v14091922)
Supplement: Supplementary file 1 [file viruses-14-01922-s001.zip › viruses-1843215-supplementary.pdf]

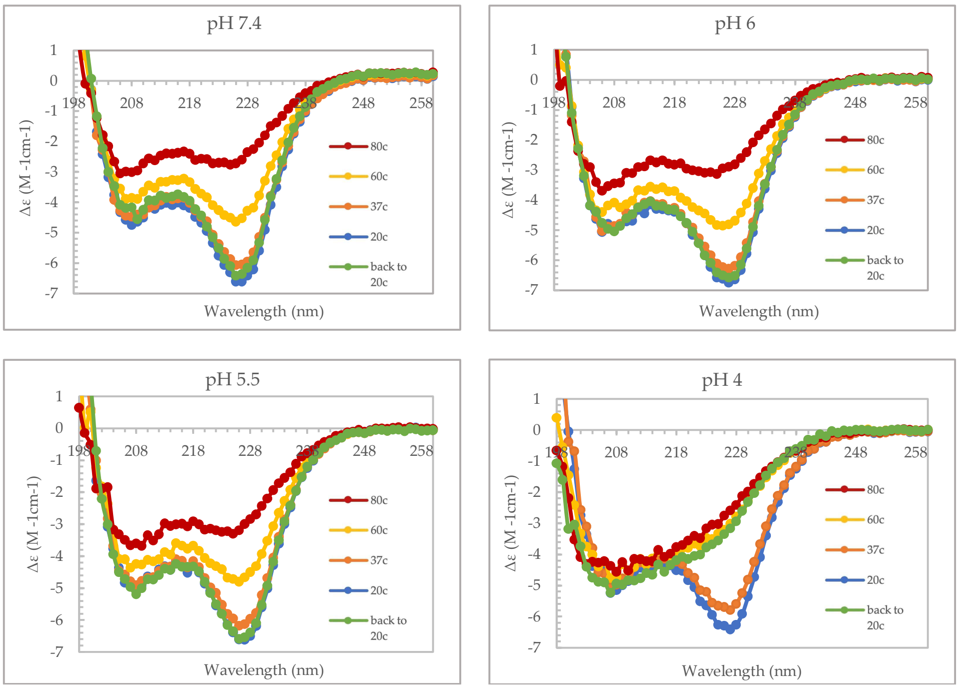

Supplementary Figure S1. Effect of heat on the secondary structure of the RBD. CD spectrum was recorded at pHs 4, 5.5, 6.0 and 7.4.
